# Wing Kinematics and Unsteady Aerodynamics of a Hummingbird Pure Yawing Maneuver

**DOI:** 10.3390/biomimetics7030115

**Published:** 2022-08-19

**Authors:** Alec Menzer, Yan Ren, Jiacheng Guo, Bret W. Tobalske, Haibo Dong

**Affiliations:** 1 Department of Mechanical and Aerospace Engineering, University of Virginia, Charlottesville, VA 22903, USA; 2 Division of Biological Sciences, University of Montana, Missoula, MT 59812, USA

**Keywords:** computational fluid dynamics simulation, hummingbird pure yaw maneuver, bio-inspired maneuvering performance

## Abstract

As one of few animals with the capability to execute agile yawing maneuvers, it is quite desirable to take inspiration from hummingbird flight aerodynamics. To understand the wing and body kinematics and associated aerodynamics of a hummingbird performing a free yawing maneuver, a crucial step in mimicking the biological motion in robotic systems, we paired accurate digital reconstruction techniques with high-fidelity computational fluid dynamics (CFD) simulations. Results of the body and wing kinematics reveal that to achieve the pure yaw maneuver, the hummingbird utilizes very little body pitching, rolling, vertical, or horizontal motion. Wing angle of incidence, stroke, and twist angles are found to be higher for the inner wing (IW) than the outer wing (OW). Unsteady aerodynamic calculations reveal that drag-based asymmetric force generation during the downstroke (DS) and upstroke (US) serves to control the speed of the turn, a characteristic that allows for great maneuvering precision. A dual-loop vortex formation during each half-stroke is found to contribute to asymmetric drag production. Wake analysis revealed that asymmetric wing kinematics led to leading-edge vortex strength differences of around 59% between the IW and OW. Finally, analysis of the role of wing flexibility revealed that flexibility is essential for generating the large torque necessary for completing the turn as well as producing sufficient lift for weight support.

## 1. Introduction

The maneuvering characteristics of natural fliers are both effective and stunning. The ability to perform agile motions, such as a pure yaw turn, is highly desirable for bio-inspired micro-aerial vehicles (MAVs). To operate in close-quarters environments and safely navigate around obstacles, the ability to change direction within only a few wingbeats is very beneficial to the performance of the vehicle. These high-agility motions can be performed by smaller creatures such as insects and even larger animals such as hummingbirds [1,2,3], and efforts of researchers have produced MAVs inspired from their wing flapping motions [4,5,6]. Hummingbird yaw turn performance differentiates from other species that perform yaw turns, such as fruit bats, hawkmoths, and fruit flies, due to active torque generation allowing for higher yaw rates achieved in a relatively low number of wingbeats [7]. While robotics works have addressed yaw capabilities of hummingbird-like flapping platforms with passive mechanisms, the real hummingbird wing motions and deformations, which could improve on the current state of performance, has not been thoroughly investigated [8].

Progress has been made to better understand the unsteady flight capabilities of insects. Study of free maneuvering of dragonflies revealed sweep speed and angle of attack asymmetries between the inner and outer wing resulting in force differences between inner wings/outer wings and forewings/hindwings [9]. For dragonfly and damselfly flight, leading-edge vortex (LEV) formation enhancements and diminutions caused by asymmetric wing kinematics produce the torque necessary to complete more intricate flight motions [10,11]. Further, fruit fly maneuvering analysis showed that asymmetric wing kinematics were needed to damp the turning motion [12]. Despite thorough investigation of insect maneuvering, in-depth analysis of hummingbirds exists primarily for forwards and hovering motion. Although insects are able to perform maneuvers, the more complex bio-mechanical characteristics of hummingbird wings may present advantages such as more control of wing kinematics leading to counter-torque generation, or torque opposite the body turning direction [7,13].

Experimental studies of hummingbirds during hovering and forward flight have informed researchers on many kinematics and aerodynamic properties. In-depth wing deformation analysis has shown that wing twisting angles increase further along the span during hovering flight [14]. Aerodynamic measurements of the wake of hovering rufous hummingbirds using digital particle image velocimetry (DPIV) have been performed too [15,16]. Results reveal evidence of LEVs over the wing surface for lift production, which is a widely documented lift mechanism in insect flight [17,18]. The mean circulation generated during the downstroke was found to be about 2.1 times of that during the upstroke. In addition, the observed 2D flow fields showed strong downwash patterns during the downstroke. Another study employed DPIV to attain detailed horizontal flow measurements of hovering Anna’s hummingbirds, which revealed distinct vortex loops underneath the wings [19]. Extending from hovering analysis, investigation of three-dimensional kinematics for forward flight of rufous hummingbirds in a wind tunnel has been conducted. Alterations in kinematics such as chord angle, angle of attack and wingbeat amplitude were observed with different incoming velocity flows [20]. Other behavior such as backward flight has also been studied [21].

In addition to these experimental studies, numerical simulations have been performed for a ruby-throated hummingbird in hovering flight at a Reynolds number of 3000 [22,23]. Similar as observed in [16], the lift production during the downstroke is about 1.5 times as much as that in upstroke, which can be attributed to the high wing angle of attack, drag-based force, and wing–wake interaction [22]. Quantification of the performance of a similar hummingbird model was achieved using a quasi-steady model. It was also found that pitch reversal of the hummingbird wing is driven by the wing inertia with no increase in power requirement due to elastic energy storage at the shoulder joint [23]. This low-fidelity model predicted overall lift production and was consistent with CFD results; however, the more simplistic methodology fails to capture detailed force oscillations associated with the highly unsteady flapping motions [24]. Numerical investigation of wing–body interaction in hummingbird forward flight revealed 29% lift enhancement due to strengthened LEVs near the wing roots [25]. However, this effect was observed to be negligible in hovering flight [16]. The previously mentioned studies do not consider how asymmetric wing motions and body rotations impact the performance and wake structures.

Laboratory tests have shown that hummingbirds can achieve sustained yaw turns by altering wingbeat kinematics: however, these hummingbirds were coaxed into performing the yaw by a revolving feeder, and the free maneuver was not thoroughly investigated [26,27]. Study of escape maneuvering body and wing kinematics have also been performed with an associated analysis of the aerodynamic performance, such as force and torque generation, in the context of controlling a maneuver [28,29,30]. The aerodynamic performances were based on a quasi-steady model, which lacks the ability to accurately capture the unsteady vortex dynamics that are exhibited by hummingbird’s wing flapping. In general, prevalence of studies addressing hummingbird free maneuvering kinematics and high-fidelity aerodynamics analysis is low despite the desirable nature of incorporating such agility into bio-inspired MAVs. The unsteady flapping and complex geometry and kinematics of hummingbird wings associated with body maneuvering warrant approaches that utilize more advanced computational modeling techniques.

In this study, reconstruction techniques are used to reveal hummingbird wing kinematics and create a realistic digital hummingbird model performing a pure yaw flight. The aerodynamic performance is then studied using an in-house developed parallel immersed boundary method (IBM)-based CFD solver. In doing so, this will reveal important aerodynamic and kinematics characteristics of the pure yaw maneuver, which hummingbird-like MAVs can take advantage of. Section 2 addresses the methodologies of this study including experimental, reconstruction, and simulation procedures. Section 3 presents findings on the wing kinematics, force generation, and wake topology. Conclusions are summarized in Section 4.

## 2. Materials and Methods

### 2.1. Experimental Setup and Procedure

Calliope hummingbirds (*Stellula calliope*) (body mass 2.6 g, Table 1) were captured from the wild under permits from the US Fish and Wildlife Service and Montana Department of Fish, Wildlife, and Parks. Approval was gained by the University of Montana Institutional Animal Care and Use Committee for the housing and experimental procedures used in the study. During captivity, birds were individually housed in 1 m × 1 m × 1 m flight cages with ad libitum access to food and water in the form of Nektar-Plus (NEKTON^®^; Günter Enderle, Pforzheim, Baden-Württemberg, Germany) or a 20% sucrose solution. The morphology of the birds was measured using techniques described in Tobalske et al. [31]. To calculate average wing chord (mm), the wing area was divided by wingspan. Aspect ratio is defined as single wing length divided by average wing chord. To determine the disc loading (N m^−2^), body weight was divided by disc area (*S_d_*). For this study *S_d_* = π(R/2)^2^, where R represents the wingspan.

We measured a yaw maneuver of a male calliope hummingbird flying inside a flight chamber (30 × 50 × 50 cm) made of open mesh supported using brass rods. The maneuver was volitional, after a bout of feeding, and not a startled response. Of the collected footage one video, in which the yaw turn was captured, is used for the body and surface reconstruction. We used three synchronized high-speed video cameras, one SA3 and two PCI-1024 (1024 × 1024 pixel resolution) (Photron Inc., San Diego, CA, USA), sampling at 1000 frames s^−1^ and with a shutter speed of 1/10,000 s. To illuminate the experimental chamber, 4 × 650 W halogen lights (Lowel Tota-light, Lowel-Light Manufacturing, Brooklyn, NY, USA) and 2 × 50-W LED lights (Fancier LED500, Ningbo Fancier Photographic Equipment Co., Zhenhai, Ningbo, China) were placed on the outside of the flight chamber. Cameras were calibrated using a direct linear transformation for three-dimensional kinematic reconstruction [32]. For other experiments, we marked the bird using 1.5-mm dots of non-toxic, water-soluble white paint.

### 2.2. Wing and Body Surface Reconstruction

**Figure 1:**
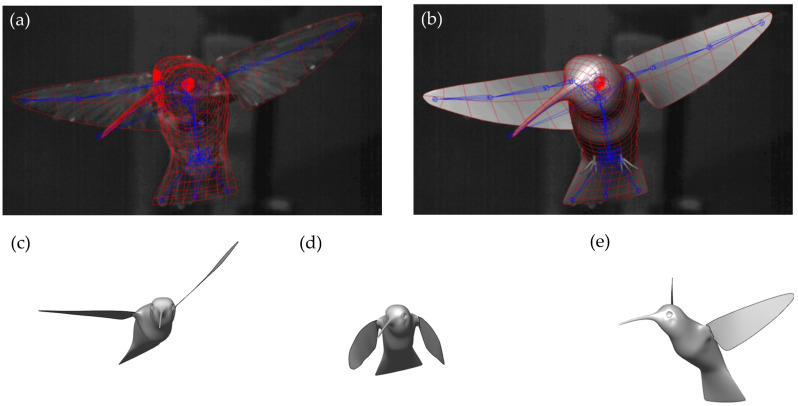
(**a**) Hummingbird skeleton and Catmull–Clark subdivisions superimposed on the real hummingbird; (**b**) fully rigged hummingbird model with skin surface; illustration of the hummingbird yaw turn at 3 time instances (**c**) 1 ms; (**d**) 26 ms; (**e**) 54 ms.

The hummingbird’s maneuvering flight was reconstructed using a joint-based hierarchical surface subdivision method. This method of reconstructing biological motion has been implemented in previous studies of tuna finlets and manta ray flapping, models that feature complex biological bodies and membranes [33,34]. The details about this method of reconstruction and its accuracy can be found in Koehler et al. [35]. A fully rigged hummingbird template with virtual joints was built based on anatomical structure of the hummingbird wing and body, as seen in Figure 1a. Manipulation of the virtual joints about the 6 degrees of freedom (translations and rotations) achieves the accurate reconstruction of flapping. The virtual joints in the wings are primarily rotated to achieve the motion, whereas the small side-to-side and up-and-down motions of the body are imposed by translating the hierarchal ‘parent’ joint of the virtual skeleton. The skin, which is bound to the skeleton, is generated with Catmull–Clark subdivision surfaces. These surfaces are spline representations of an arbitrary mesh topology and thus are suitable for modelling the hummingbird body and wing surface [36]. A rendering of these surfaces can be seen as the red meshes in Figure 1a,b. The wings are modeled as separate surfaces to the body surface but are attached continuously to the body at the wing roots. In Figure 1c–e, 3-time instances of the turning flight are shown (1 ms, 26 ms, and 54 ms). In total, the hummingbird maneuver lasted 113 ms from the start of the downstroke of the 1st stroke to the top of the upstroke of the 6th stroke.

**Figure 2:**
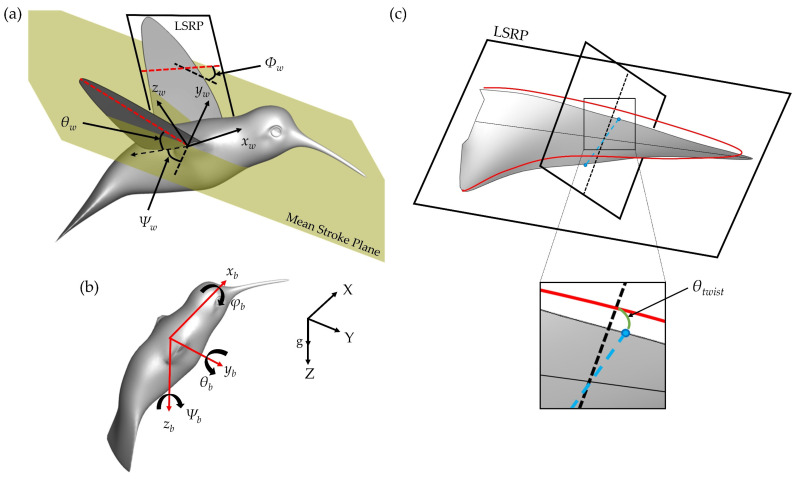
(**a**) Illustration of the wing kinematics angles of stroke *Ψ_w_*, deviation *θ_w_*, and pitch *Φ_w_*. The wing root-to-tip vector is shown as a dotted red line, the wing leading-edge to trailing-edge vector is shown as a dotted red line on the opposing wing; (**b**) Visualization of the body coordinate system with global reference frame pictured; (**c**) LSRP rendering with definition of *θ_twist_* as the angle between the wing and the least deformed wing plane.

Figure 2a illustrates the three wing position angles relative to the wing–root coordinate system (*x_w_ y_w_ z_w_*), in which the *x_w_*-axis is parallel with the body longitudinal direction (or root-to-root), the *y_w_*-axis is along the lateral direction, and the *z_w_* axis complies with the right-hand rule. The mean stroke plane (MSP) serves to indicate the plane formed by the average of the tip positions at the top and bottom of the stroke as well as the wing root. The stroke position angle *Ψ_w_* defines the forwards or backwards position relative to the *y_w_* axis. The dotted black arrow in the stroke plane represents a projection of the tip to root vector (red vector) onto the stroke plane. To measure the upwards and downwards position with respect to the MSP, deviation angle *θ_w_* is used. *θ_w_* represents the angle between the base-to-wingtip line and the root–tip vector projection. The angle of incidence (AOI) *Φ_w_* is defined as the angle between the least-squares reference plane (LSRP) and the tangent of the wing velocity in the MSP. To compute the LSRP, the displacements of the wing surface nodes relative to a flat wing in the least-squares plane (red outline in Figure 2c) are minimized [30]. With the LSRP, the twisting angle *θ_twist_* is defined as the measure of the angle between least-squares wing (red) and deformed (grey) wing. This is illustrated in Figure 2c. This measurement is useful in determining the spatial and temporal variations of wing twisting deformations. Figure 2b illustrates the body coordinate system (*x_b_ y_b_ z_b_*) with yaw, pitch, and roll identified as well as the global coordinates.

### 2.3. Numerical Methods and Simulation Setup

The governing fluids equations solved in this work are the incompressible Navier–Stokes equations shown in indicial form in Equation (1). In Equation (1), *u_i_* are the velocity components, *p* is the pressure component, and *Re* is the Reynolds number. This non-dimensional value is computed via $Re=\frac{2MRfc}{\nu }$ [22] where *M* is the flapping angle amplitude, *R* is the wing length, *f* is the flapping frequency, *c* is the chord length, and ν is the kinematic viscosity of air. For the current study, the Reynolds number is selected to be 3640, which falls within the range of Reynolds numbers for other numerical studies of hummingbird flight [22,23].

(1) $$\frac{\partial u_{i}}{\partial x_{i}}=0;\;\frac{\partial u_{i}}{\partial t}+\frac{\partial u_{i}u_{j}}{\partial x_{j}}=-\frac{\partial p}{\partial x_{i}}+\frac{1}{Re}\frac{\partial^{2}u_{i}}{\partial x_{i}\partial x_{j}}$$

The above equations are discretized using a cell-centered, collocated arrangement of the primitive variables (*u_i_*, *p*). In addition to the cell-centered velocities (*u_i_*) that satisfy the momentum equations, the face-centered velocities, which satisfy mass conservation, are also computed [37]. Additionally, all length and time scales are in their appropriate dimensionless forms. A second order accuracy fractional-step method is used for the time-advancement of the above equation. Convective terms in Equation (1) are discretized using second-order accurate Adams–Bashforth scheme, while a second-order accurate Crank–Nicolson scheme is employed for the diffusion terms in Equation (1). The current computational method has been employed to study the unsteady aerodynamics of butterfly flapping, flexible wings, hovering flight, and cicada flight [38,39,40,41], which are closely related to the hummingbird flapping in the current study. Validations of the solver for complex biological boundaries can be found in [25,42,43]. More details on the numerical algorithm and immersed-boundary treatment, as well as validations, can also be found in Mittal et al. [44].

**Figure 3:**
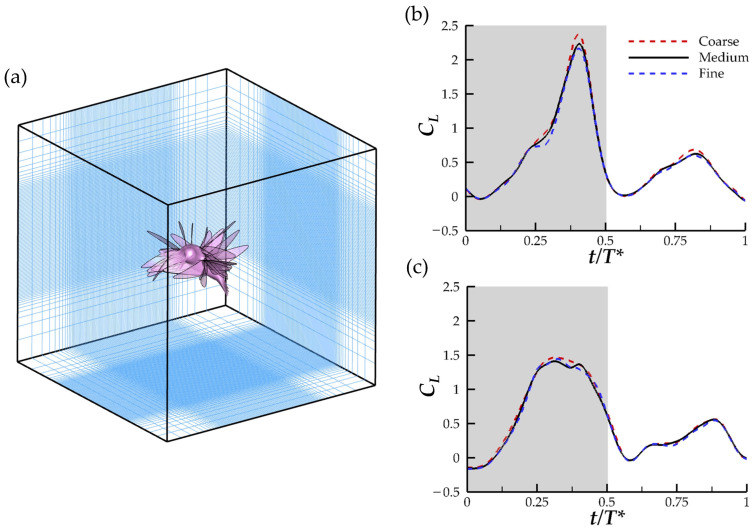
(**a**) Computational domain illustrating the orientation of the body in the fluid domain, (**b**) comparison of the forces for the inner wing and (**c**) outer wing for the coarse mesh (~7.1 million grids), medium mesh (~11.2 million grids), and fine mesh (~17.0 million grids).

The computational domain has dimensions of 20 × 20 × 20 c. A non-conformal Cartesian grid configuration with a stretching region surrounding the densest grids is used. On each of the domain boundaries, all velocity gradients and pressure gradients are set to zero. For the initial conditions of the simulation, no flow speed is prescribed as the reconstructed model performs the motion of a yawing hummingbird in a still-air environment. Figure 3a shows the computational domain and positioning of the hummingbird. To solve the governing fluids equations on the computational domain, 4 CPU cores were deemed sufficient. Using the University of Virginia Research Computing groups high performance computing cluster, Rivanna, the simulation of the hummingbird maneuver cases took about 160 wall clock hours or 640 CPU hours.

The grid spacings were determined using a grid-independent study in which meshes with 3 different grid densities are computed: coarse (∆ = 0.056 c), medium (∆ = 0.045 c), and fine (∆ = 0.038 c). The lift force produced by the hummingbird model during the 3rd stroke motion is compared. This stroke best approximates the average kinematics and force generation during the turning motion. Comparison of the resultant force produced by the inner and outside wing can be found in Figure 3b,c, respectively. The maximum inner wing (IW) *C_L_* for the medium mesh varies from the coarse mesh by 5.3% and fine mesh by 1.8%. For the outer wing, the variation from the medium mesh is 5.1% and 2.1% for the coarse and fine mesh, respectively. Thus, the medium mesh is determined to be of sufficient fine-ness for the current study.

## 3. Results and Discussion

We first report the body and wing kinematics of this flight. This is followed by the results of aerodynamic forces produced by the wings. Next, the three-dimensional flow structures in the far and near wake are explained. By simulating the flow over the six stroke motions performed by the hummingbird, results on the aerodynamic force and wake topology are obtained. Following this, evaluation on the role of wing flexibility in performing the maneuver is conducted.

### 3.1. Body and Wing Kinematics Measurements

**Figure 4:**
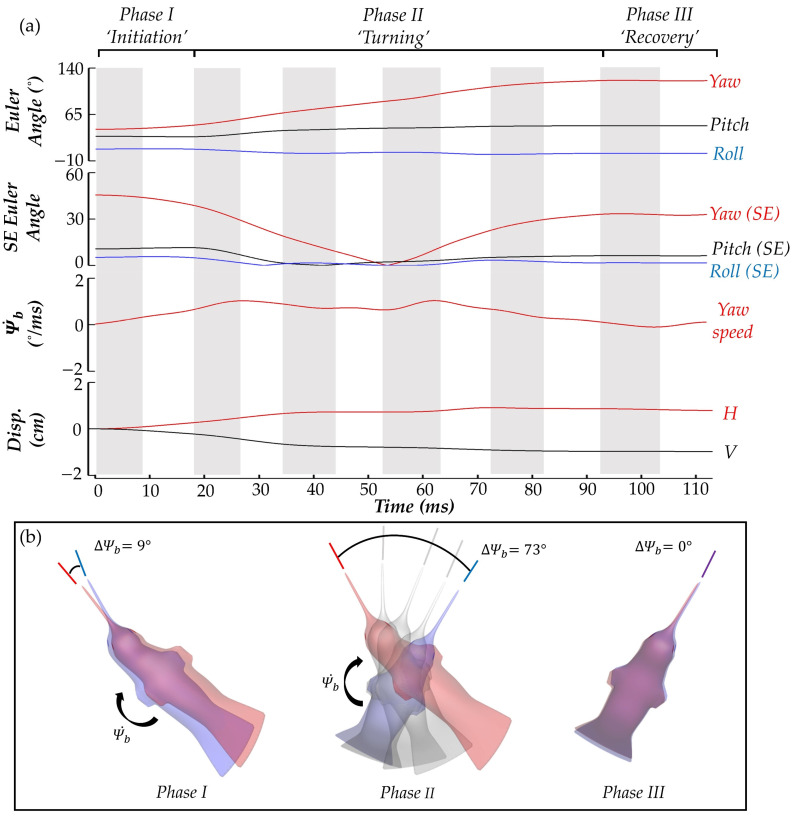
Summary of the body kinematics of the hummingbird including (**a**) body Euler angles, root squared error of the body Euler angles relative to the mean, plot of yaw angular velocity, and body horizontal (H) and vertical (V) displacements as well as (**b**) visualization of the yaw turn.

Findings on the body and wing kinematics of the hummingbird performing a yaw turn are presented in this section. First the body kinematics, including the time series of the body yaw, pitch, and roll angles as well as yaw velocity and body position, are displayed in Figure 4a. During the maneuvering process, the hummingbird performs six flapping motions in total. The downstrokes and upstrokes are differentiated by the coloring of the plot background with grey indicating the downstroke (DS) motion and white indicating the upstroke (US) motion. To better interpret the characteristics of the turn, the process is divided into three phases according to the body yaw angle and yaw velocity.

During phase I, the yaw velocity monotonically increases, and the yaw angle increases slightly (+9°). Phase II is characterized by a sustained increase in yaw angle (+73°) and an oscillating yaw velocity profile. This oscillating profile indicates that the hummingbird is actively controlling its orientation during the turn. Finally, during phase III, the hummingbird yaw angle increases negligibly (0°), and the yaw velocity is close to 0. From the root square error relative to the mean body Euler angle plot, the only body angle that changes considerably throughout the motion is the yawing angle (red), as the pitch and roll (black and blue) vary minimally about each respective mean value. Thus, the motion is termed a pure yawing turn.

In Figure 4b, top-down views of the hummingbird body at the start (red) and end (blue) of each phases I-III are shown to visualize the progression through the yaw motion. The angular change in body yaw is visualized by lines pointing in the heading direction of the hummingbird at the start (red) and finish (blue) of the phase. The light grey bodies and heading direction lines indicate the body position at the end of each of the middle strokes during phase II. Next, the wing kinematics are presented.

**Figure 5:**
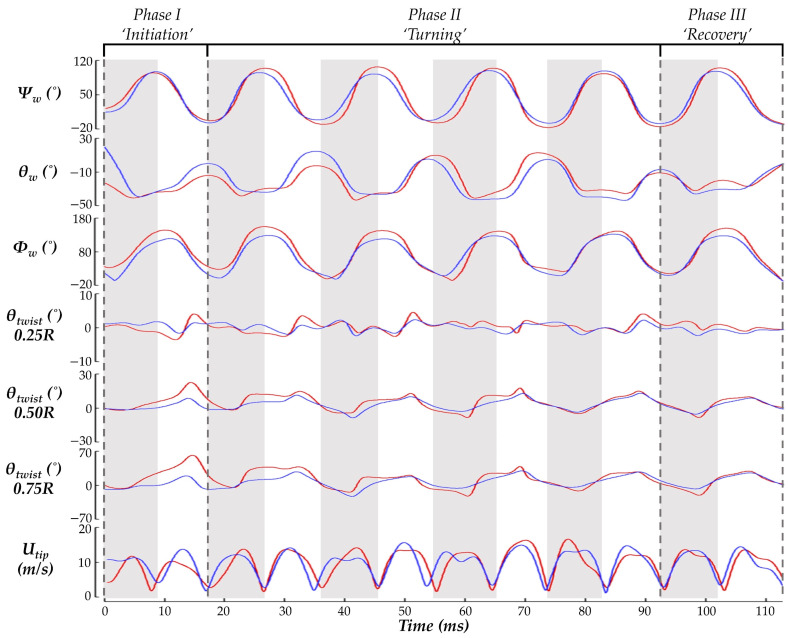
Wingbeat kinematics variables of stroke (*Ψ_w_*), deviation (*θ_w_*), angle of incidence (*Φ_w_*), *θ_twist_* at 0.25 R, 0.50 R, and 0.75 R, and wing tip velocity (*U_tip_*) during the entire maneuvering motion. DS is indicated by grey shading and US is indicated by white shading. IW is indicated by red curves and OW is indicated by blue curves. R represents span length.

Figure 5 displays time series of the wing kinematics *Ψ_w_*, *θ_w_*, *Φ_w_*, and *θ_twist_*. During the pure yaw maneuver, the hummingbird flapped with considerable angle of incidence, stroke, and twisting angle asymmetries. A quantitative comparison between the wing angle amplitudes is given in Table 2. This helps to understand how the hummingbird expands or suppresses its wing kinematics to perform the maneuver. To initiate the turn, a suppression of the IW deviation and stroke angle is observed. During the turning phase, the IW maintains an expanded stroke and AOI amplitude compared to the OW, but the ‘figure eight’ shape of the IW is smaller than that of the OW as indicated by the smaller deviation amplitude. During the recovery, in which hovering is resumed, the wing kinematics for the IW and OW are similar with the only considerable asymmetry present for the AOI.

For twisting, chords closest to the wing root experienced the least amount of deformation (within ±5° at 0.25 R). The twist angle amplitude rapidly increases further out on the span with the large deformations occurring at 0.75 R, which is consistent with previous measurements [14]. However, the IW and OW asymmetries observed in this study are not seen in other measurements. Here, the IW twists nearly perpendicular to the LSRP during stroke I. Subsequently, the deformations stabilize to a more repetitive nature in which the IW typically exceeds the OW twist. Further out along the wing, at 0.75 R, the square-like profile of the IW twisting angle leads to sustained higher twist during the US. Consequently, the average IW twist during the US is higher than the OW by 21.3% at 0.5 R and 46.4% at 0.75 R, suggesting the hummingbird asymmetrically attenuates and expands its wing twisting for the IW and OW to achieve the turn.

Finally, the wing tip velocities are compared. During stroke I, the OW *U_tip_* is much higher than that of the IW during the US, which corresponds with the initiation of the yawing motion. During the subsequent DS for strokes II-V, the IW tends to have a higher velocity whereas during the stroke II-V US, neither the IW nor the OW tip velocity is dominant. Interestingly, the OW tip velocity tends to exhibit a plateau during the DS while the IW continues to speed up until reaching a peak velocity at the mid-stroke.

Compared to previous works involving hummingbird turning, the body kinematics identified for the current calliope hummingbird present key differences. Use of revolving feeders limits the turning speed of hummingbirds to around 0.5 Hz to 0.6 Hz at maximum [26,27]. The data collected in the current study, in which 82° are traversed in 113 ms, extrapolates to 2.01 Hz, which falls within the range of the free maneuvering turning speed discussed by Altshuler et al., suggesting that the coaxing mechanism constrains the turning performance [26]. Additionally, the coaxed-turn measurements revealed body banking that is absent in the current free maneuvering hummingbird [27].

Wing kinematics for the free maneuvering hummingbird also vary from coaxed-turn measurements. Namely, the stoke angle amplitudes observed in the current work are smaller with the primary difference arising due to suppressed maximum US stroke angle. Additionally, larger DS elevation angle amplitude is achieved [26,27]. However, these wing kinematic findings do agree with the general trends observed by Read et al., where an increase in yaw rotational speed corresponded with an increase in DS elevation angle. As the turn speed of the hummingbird examined in the current work is nearly four times greater than that in Read et al. [27], the large DS elevation angle amplitude is within reason. Association of the stroke amplitude is less clear, although hummingbirds often use stroke amplitude changes to manage aerodynamic power, and the shrunken amplitude in the current work may indicate lower power consumption during the turn [18,45,46].

### 3.2. Wing Aerodynamic Forces

To better understand how the previously discussed wingbeat kinematics are utilized by the hummingbird to perform the yawing maneuver, the results of the aerodynamic forces and power consumption are shown. Forces are obtained by integrating the pressure and shear stress over the surface of the wings. The forces are resolved into two components: along the global-Z direction, and in the global XY plane opposite to the hummingbird heading direction. These forces are termed lift (*F_L_*) and drag (*F_D_*), respectively. *Power* is computed by summing the velocity of the surface element (relative to the local fluid velocity) multiplied by the force on that element across all the wing surface elements.

**Figure 6:**
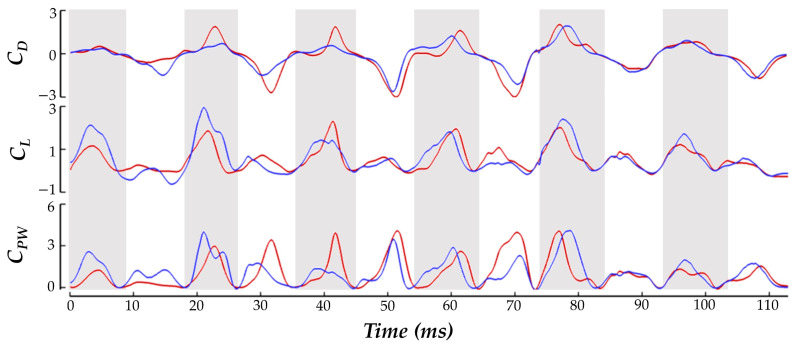
Time series comparison of the normalized drag, lift, and power consumption by the inner (red) and outer (blue) wing.

The force and power are plotted as *C_D_*, *C_L_*, and *C_PW_*, which are non-dimensional and normalized values determined by:

(2) $$C_{D,L}=\frac{F_{D,L}}{\frac{1}{2}\rho U_{tip}{}^{2}S_{b}},\;C_{PW}=\frac{Power}{\frac{1}{2}\rho U_{tip}{}^{3}S_{b}}$$

where *F_D_*_,*L*_ indicates drag and lift force, respectively, *U_tip_* represents average tip velocity, and *S_b_* is the wing surface area. The hummingbird force production during the pure yaw maneuver is shown in Figure 6 as the time series of *C_D_* and *C_L_*. Under the current convention, a positive drag force generated by the inside wing would point backwards relative to the hummingbird heading and create a clockwise moment about the body.

The balancing of *C_D_* generation to achieve the yaw turn is clear. During both the US and DS, the IW achieves about 16% higher magnitude drag force than the OW in total. The direction of the drag force alternates in such a manner that the DS produces positive drag (opposite to the heading direction), and the US produces negative drag (towards the heading direction). This means that the higher magnitude IW drag forces serves to drive the clockwise turn in the DS and damp the turn in the US, which helps to explain the yaw velocity oscillations discussed previously. For the IW, the US contributes about 69% more drag generation than the DS. For the OW, the US accounts for 80% more drag generation than the DS. The OW only consistently exceeds the drag production of the IW in the stroke I US where the drag asymmetry favoring the OW aids in initiating the clockwise turn. During stroke VI, the recovery phase, the drag forces are the same for both the IW and OW. This matches exactly with the period in which the yaw turns slow and hovering resumes.

For the lift production, the IW and OW remain relatively similar across the whole motion with no consistent bias towards lifting force on one wing. The mostly positive *C_L_* means that the hummingbird US and DS both generate upwards force opposing gravity which is likely a mechanism adopted by the hummingbird to maintain level vertical displacement during the motion (as observed in the body kinematics analysis). Further examination reveals that the DS produces significantly higher lift than the US. Averaging between the IW and OW, the DS generates 73% more lift than the US throughout the whole maneuver which is consistent with previous studies on hummingbird flight aerodynamic force generation for hovering flight [15,16,22]. Using the hummingbird mass (2.6 ± 0.2 g, Table 1), and the gravitational acceleration *g* = 9.81 m s^−2^, the force required to keep the hummingbird aloft is about 2.5 × 10^−2^ ± 0.2 × 10^−3^ N. The current *C_L_* definition defines lift to be opposite to gravity. Re-dimensionalizing the current results reveals that the average lift force produced during the maneuver is 2.3 × 10^−2^ N. This agrees well with the force necessary to keep the hummingbird aloft with minimal elevation change, which was observed in the body kinematics in Figure 4a.

Discussing power, to initiate the turn in stroke I the hummingbird expends 153% more power flapping the OW compared to the IW. During the turning phase the power consumption for the IW has higher peaks than the OW at the mid-DS/US. During the resumption of hovering flight in phase III, power consumption is significantly reduced for both wings, and no IW/OW discrepancies are seen.

**Figure 7:**
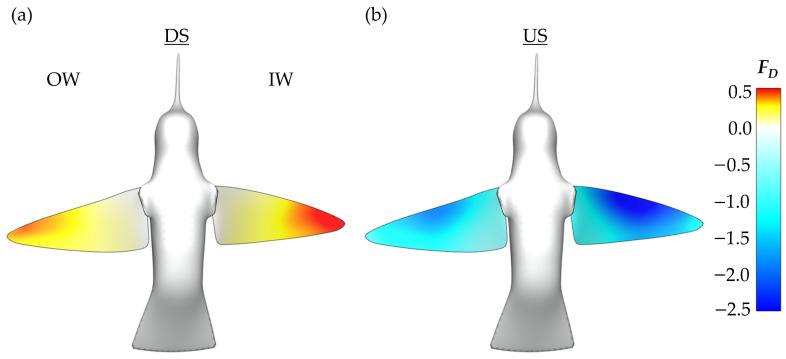
(**a**) Phase II, ‘turning phase’, average DS and (**b**) average US non-dimensional drag forces produced by the wings.

In addition to the time series data presented in Figure 6, plots depicting the US and DS average forces during phase II are provided in Figure 7. The forces are plotted on the model whose position allows for clear visibility of both wings. This serves to further clarify the role of the half strokes in force generation and visualize the asymmetric aerodynamic forces. The DS average, shown in Figure 7a, illustrates that drag generation is primarily concentrated near the wing tips. Further, the IW has more intense drag generation as indicated by the darker red regions. Thus, the yaw turn is enhanced by the DS. For the US average in Figure 7b the magnitudes of the averaged drag force are larger than the DS. The IW again demonstrates increased drag production compared to the OW as seen by the larger dark blue region near the mid-span. However, the torque generation during the US is opposite that of the DS as the IW biased drag production serves to damp the yaw turn.

### 3.3. Turning Motion Wake Topology

In this section, the arrangement and timing of vortex production and shedding is examined. The snapshots of wake topology are provided for the third flapping motion during which the hummingbird is performing its turn. This time frame was selected since the hummingbird has settled into the steady yaw angle increase. Additionally, the forces during this stroke closely match the overall trends noticed in the previous section (i.e., the larger IW US/DS *C_D_* as well as significant DS *C_L_* generation). Therefore, stroke 3 will serve as a good estimate for the average wakes produced during the turning motion.

To understand the organization of the vortex structures, iso-surfaces visualized with a *Q*-criterion value of 375 are displayed. The *Q*-criterion, which is dimensionless in nature, is calculated according to Equation (3) where $\Omega =\frac{1}{2}\left[\nabla u-\nabla u^{T}\right]$ and $S=\frac{1}{2}\left[\nabla u+\nabla u^{T}\right]$ are the vorticity and strain rate tensors, respectively. This variable is helpful in identifying rotationally dominant regions of the fluid.

(3) $$Q=\frac{1}{2}\left[{\left|\Omega \right|}^{2}-{\left|S\right|}^{2}\right]$$

**Figure 8:**
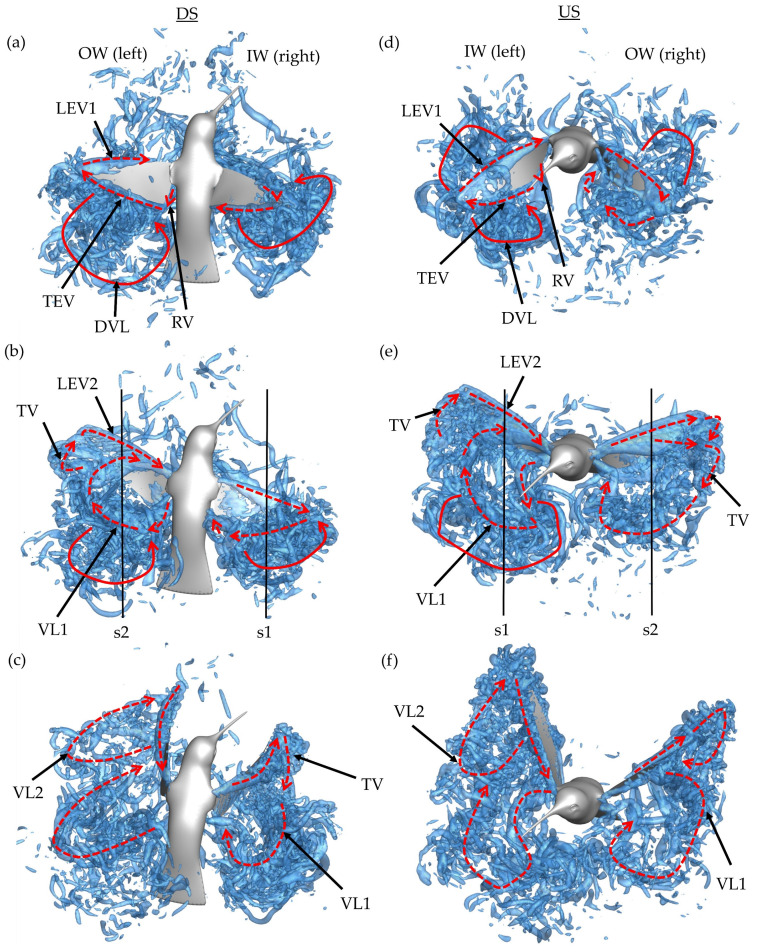
Wake topology generated by the hummingbird’s third DS at (**a**) t/T* = 0.16, (**b**) t/T* = 0.32, and (**c**) t/T* = 0.48 and third stroke US at (**d**) t/T* = 0.60, (**e**) t/T* = 0.72, and (**f**) t/T* = 0.98. The lines labelled s1 and s2 in (**b**,**e**) denote the slices that the data in Figure 9 are shown on.

The downstroke motion is analyzed first in Figure 8a–c. Due to the orientation of the body and position of the wings, the images are shown from above and behind the hummingbird. This perspective allows for clearer differentiation of the downstroke wake topology. Plots for the DS are shown at t/T* = 0.16, t/T* = 0.32 and t/T* = 0.48. The time stamp t/T* = 0.32 corresponds closely with the time at which maximum DS force is achieved. T* stands for the time required to complete a stroke motion. Newly formed vortices are highlighted with dotted red arrows while vortex loops generated during the previous half-stroke are highlighted in solid red.

Early in the DS, formation of the leading-edge vortex (LEV1), trailing-edge vortex (TEV), and root vortex (RV) is observed on the OW in Figure 8a. At this time, a major discrepancy between the IW and OW is present. The OW LEV1 occupies a large portion of the wing leading edge, with more chordwise expansion towards the distal regions, meanwhile the IW LEV1 is comparatively smaller. In addition to the newly created structures, the detached vortex loop (DVL) from the previous upstroke can be seen above and behind both the IW and OW.

At the mid downstroke, shown in Figure 8b, the outer wing exhibits a dual-vortex loop formation. The inner-most loop, labeled VL1, forms because of early LEV1 lift-off from the wing and is composed of the detached arch-like LEV1, TEV, and RV. Connection between the previously formed RV and TEV is clear. The outer-most loop, labeled VL2, starts to form between the tip vortex (TV) and newly generated LEV2; however, no clear ring-like structure is seen. The dual loop generation is like that observed with AR 2,4 revolving plates in DPIV experiments and simulations [47,48,49]. For the wings of the current computational model, AR ~3. The inner wing exhibits a more uniform shedding pattern than the outside wing with the LEV1, TEV, and RV forming a single loop much like the topology documented in ruby-throated hummingbird hovering flight [22].

Finally, the end of the downstroke is shown in Figure 8c. The previously visible VLs (both for the inner and outer wing) have dissipated. Behind the outer wing, the dual loop vortex pattern is more visible as the TV in VL2 has stretched due to the wing flapping motion. Meanwhile, VL1 has mostly separated from the wing and is positioned behind the wing. As for the inside wing, the coherent VL1 is still prevalent with the TV stretching to connect the VL1 to LEV1. This end-stroke vortex organization for the IW is like the wake of a hovering ruby-throated hummingbird. Song et al. observed the TV connecting the TEV to the primary LEV at the end of the hovering DS at *Re* = 1500 [22].

Next, the wake topology of the upstroke phase is examined. Plots for the upstroke are shown at t/T* = 0.60, t/T* = 0.72 and t/T* = 0.88. Peak US force generation is captured at the t/T* = 0.72 frame. A very similar process to the downstroke is repeated; however, the wing on which the dual vortex loops is formed is now the inside wing (while this process also occurs on the OW, it is later in the US). This is seen in Figure 8d–f where the pattern of vortex formation follows closely with that described for the downstroke. The LEV1 formed on the IW detaches before the half downstroke causing VL1 and VL2 formation in Figure 8d. Meanwhile, the OW demonstrates more an expanded LEV1 formation, with Figure 8e showing significant chordwise expansion, much more than demonstrated by the IW during the DS. This instant captures the onset of the LEV separation. At the conclusion of the US in Figure 8f, the generated VLs are visible in the wake. Two distinct loops are visible for the OW, with the size of VL2 being small, indicating that LEV splitting occurred later in the US compared to the IW.

It appears that the main mechanism behind the dual-vortex loop vortex formation is destabilization of the primary formed LEV1. VL1 is formed due to the LEV1, TEV, and RV ring detaching from the upper surface during the downstroke and lower surface during the upstroke. When this process occurs early in the half-stroke, a substantially sized VL2 forms due to TV and LEV2 at the distal regions of the wing. As with the OW during the US, a smaller VL2 may form with later LEV1 detaching. The dual LEV generation is documented to occur beyond the midspan for higher aspect ratio flapping plates, with the tendency of the splitting to propagate from tip to root [48,50]. During the DS, the OW motion and body turning constructively interact, giving the OW a higher effective speed, while the IW experiences the same in the US. Extrapolating on the discussion by Harbig et al., where a higher advance ratio led to unstable LEVs during the DS (wing flapping in the direction of travel), we find that the wing flapping in the direction of body rotation also experiences LEV instability [50]. This is reflected by the early half-stroke LEV1 separation during the DS for the OW and US for the IW. In addition to these wing-generated vortex structures, it is important to note that no body vortex structures, such as a head shear layer and thorax vortex, are observed, which contrasts that of hummingbird forward flight [25]. This can be attributed to the much lower translational speed of the hummingbird body as most of the motion during the maneuver is rotational.

**Figure 9:**
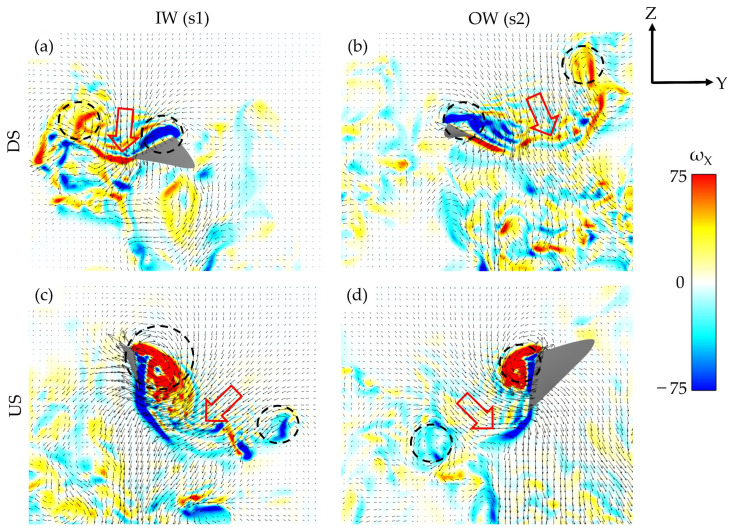
Comparison of wake jets for the IW (**a**,**c**), OW (**b**,**d**) during the DS and US, respectively.

Figure 9a–d illustrate the down jets produced by the wing flapping. Figure 9a,b correspond to the s1 and s2 locations shown in Figure 8b, while Figure 9c,d correspond to the s1 and s2 locations in Figure 8e. The vorticity contours represent the global coordinate x-directional vorticity, while the arrowheads indicate the global YZ fluid velocity components in the slice plane. The trend in DS-dominated lift and US-dominated drag production is evident in the velocity gradients. The direction of the VL jets can be thought of as a reflection of the direction of the aerodynamic forces. Figure 9a,b illustrated that the DS-generated vortex loops exhibit nearly vertical downs jets, representative of the wings generating significant lift as discussed in Section 3.2. In Figure 9c,d, the more horizontal orientations of the VL jets indicates the increased US drag generation. The general arrangement of the loops and resulting downwash agrees well with DPIV experiments and quasi-steady simulations performed on hovering hummingbirds, with discrepancies in wake direction attributed to the asymmetric flapping/body motion of the turning hummingbird [16,19,22].

### 3.4. Near-Body Vortex Structures

**Figure 10:**
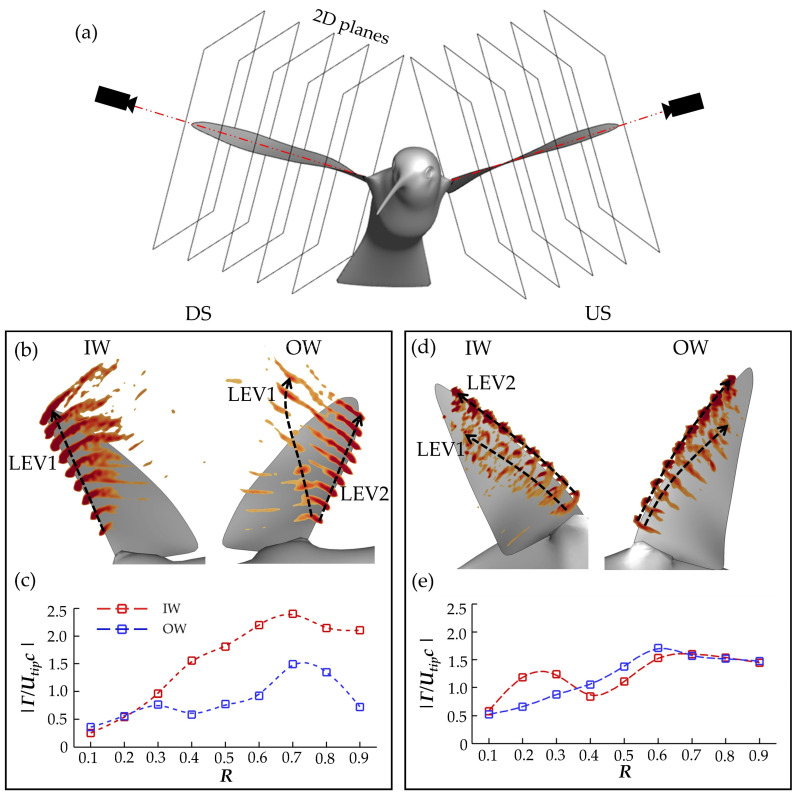
(**a**) Schematic of camera placement for computing the 2D spanwise slice cuts; (**b**) IW and OW vorticity contours at peak DS force production with (**c**) corresponding Γ, normalized by average *U_tip_c*; (**d**,**e**) represent the same information as (**b**,**c**) but for the US.

To better understand the LEV1,2 generation and destabilization dynamics, this section examines the flow field immediately surrounding the wings. To calculate spanwise vorticity of these vortexes, virtual cameras are positioned to view directly parallel to the root-tip vector of each of the wings during the flapping motion. A schematic of this is shown in Figure 10a. Slice-cuts are made perpendicular to these vectors and variables such as velocity, vorticity, and pressure in these 2D planes can be computed. This allows for calculation of circulation according to:

(4) $$\Gamma =\overset{\;}{\underset{S}{\iint }}\omega_{Z}\cdot dS$$

where *S* represents the boundary of the vortex in the 2D plane (defined by a vorticity threshold of ~15% of the maximum spanwise vorticity) and $\omega_{Z}$ represents spanwise vorticity. To minimize the erroneous inclusion of shear layers or other minor vortexes in the calculation, a search range is prescribed to the calculation so that only the region containing the LEV attached to the wing is searched. This method has been used in previous studies for measuring and comparing vortex circulation of various flying insects [9,10,11]. Stroke 3 will be examined in close detail, as with the previous section. In Figure 10b,d, slice cut vorticity along the span in increments of 0.10 R is shown at peak force production instances for the DS(b) and US(d). Additionally, Figure 10c,e compares the spanwise circulation calculation along the wing at the same instances.

Due to the early OW LEV1 separation, LEV1 is hardly visible in the slice frame. In contrast, the IW LEV is large and has more coherent dark red vorticity regions in Figure 10b. No secondary structure, either behind LEV or shed into the wake, is visible. The vorticity regions are much stronger than for the IW, too, suggesting greater LEV strength. This is supported by the Γ calculations as the peak IW Γ (at 0.70 R) is 59% greater than the peak OW Γ. Additionally, from Figure 10c, the IW Γ is much higher than that of the OW from 0.40 R outwards. For the US, the IW and OW are slightly more balanced in terms of vorticity contour and circulation strength. The multi-LEV arrangement, a consequence of early US LEV1 separation, can be seen for the IW, whereas the onset of such bifurcation is captured for the OW. The spanwise LEV2 Γ distributions are similar between the IW and OW, which generally agrees with the similar drag and lift force generation observed during the US in Section 3.2.

No direct comparisons to previous studies on turning hummingbird LEV dynamics can be made; however, works addressing revolving plates and hummingbird hovering serve as good benchmarks to discuss how the observed dynamics fit into the existing literature. From DPIV measurements, Warrick et al. concluded that LEV circulation during hovering hummingbird flight was significantly higher during the DS than US, nearly 2× [16]. Our calculation indicates the difference is 1.59×; however, the body motion and wing motion for hovering flight is different than in the current free maneuvering case, which may explain the discrepancy. Additionally, in the current study the IW LEV1 in the DS is more steadily developed, which is reflected by the strong Γ beyond the mid-span. This agrees with LEV calculations made for a revolving wing (*Re* = 1500) by Chen et al. [51]. Due to the instability, LEV2 circulation (as opposed to LEV1) is captured by the Γ calculations. Compared to the IW Γ distribution shown in Figure 10c, which represents the primary LEV1, the secondary LEV2s are weaker. Using DPIV experiments, Car et al. visualized the spanwise vorticity during the LEV1 separation, with clear inwards propagation of the split, and reported similar results. The attached LEV2 exhibited stronger vorticity regions until about 0.75 R, after which the LEV2 cross section was less organized [47]. Figure 10b,d qualitatively support this, while Figure 10c,e serve as a further quantitative validation: the LEV2 circulation decreases after about the 0.75 R span for the cases in which LEV1 separates.

**Figure 11:**
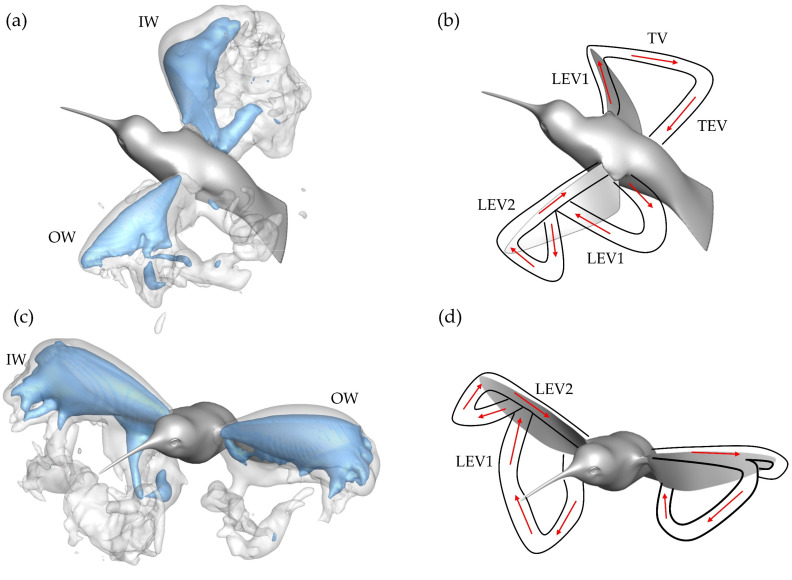
(**a**) Pressure iso-surface with (**b**) associated vortex structures generated during the DS; (**c**,**d**) represent the same information as (**a**,**b**) but for the US.

To better understand the role of the asymmetric vortex generation in force production, non-dimensional pressure iso-surfaces are plotted in Figure 1a,c. These surfaces helps to visualize the IW/OW pressure discrepancy that produces enhanced IW drag production. The blue surface indicates a non-dimensional pressure coefficient of −3.0, the white surface indicates a pressure coefficient of −1.0. This is paired with topology schematics in Figure 11b,d to help associate the pressure regions with vortex structures.

For the DS in Figure 11a,b, the IW exhibits a much stronger region of low pressure behind the wing with noticeable concentration of low pressure at the distal region of the wing due to a steadily formed LEV1. The concentration of low pressure at these positions aligns with observations of the average DS drag generation occurring mostly at the wing tips from Figure 7a. The OW has comparatively weaker regions of low pressure due to the more unsteady LEV formation. Additionally, a distinguishable VL1 that already detached from the wing is seen behind the OW. Thus, the more steadily developed and stronger LEV1 produces a more enhanced low-pressure region behind the wing leading to the higher IW drag production during the DS.

During the US in Figure 11c,d, the pressure iso-surfaces are similar between the IW and OW although the IW low-pressure region appears slightly larger than that of the OW, which leads to the observed IW drag force being larger than the OW. For the IW, this low-pressure region is associated with the LEV2 formed after LEV1 lift-off. As for the OW, the steadily developed LEV1 is responsible for the induced low-pressure region. Finally, the low-pressure region is robust even at the wing mid-span, which contrasts the DS. This matches well with the average drag force calculations illustrated in Figure 7b, where much of the US drag force is shown to be generated near the mid-span.

The dual-vortex loop formation and shedding can be summarized as the following. During the early DS, the LEV1 formed by the OW separates due to the inwards propagating ‘burst’, resulting in the formation of LEV2. Meanwhile, the IW develops a stable and strong LEV1, evident in the vorticity slice cuts in Figure 10b and circulation distribution in Figure 10c. Correspondingly, stronger low pressure is induced behind the wing, according to Figure 11a, through which higher drag is produced. The DS drag asymmetry between the IW and OW sustains the yaw turn. During the US the LEV2s, present on the IW and OW around when maximum force is generated, are similar in strength to the LEV2 on the OW during the DS (Figure 10c,d). The US low-pressure concentrations are similar too, from Figure 11c, thus there is only a slight IW drag advantage over the OW during the US, which serves to damp the body turning speed. The observed dual-ring pattern results in vortex-induced force asymmetry that contributes to the hummingbird’s ability to execute the desired yaw turn. The wake pattern led to the IW maintaining higher drag production compared to the OW, which could be due to the expanded stroke amplitudes and more suitable wing twisting for drag generation. Additionally, rotational motion of the body may contribute to the pattern [52,53].

### 3.5. Wing Flexibility

**Figure 12:**
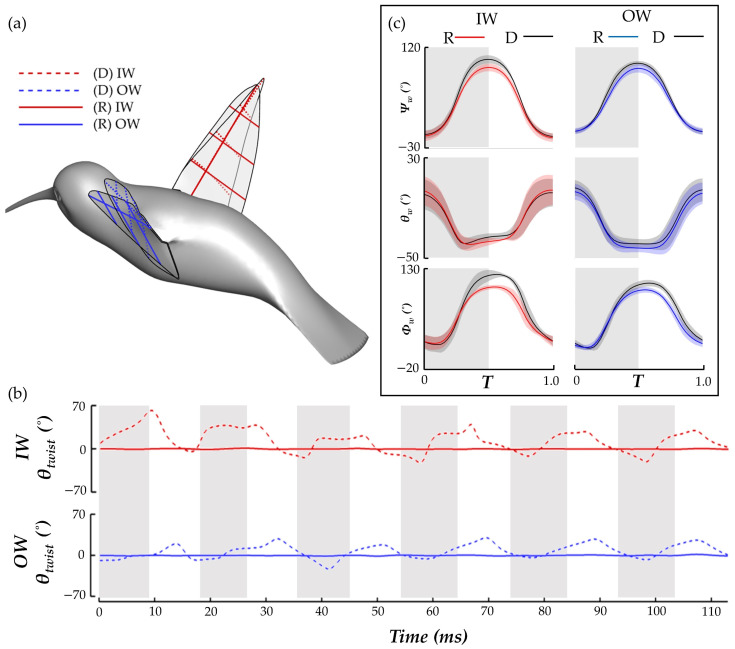
(**a**) Comparison of the deforming (dotted lines) and rigid (solid lines) wing models through tracking the chord lines at different span lengths; (**b**) quantitative comparison of IW and OW *θ_twist_* for the deforming and rigid models; (**c**) comparison of the R and D model wing kinematics of stroke (*Ψ_w_*), deviation (*θ_w_*), angle of incidence (*Φ_w_*).

Additionally, it is important to understand the role of wing flexibility in the maneuver. To achieve this, the nominal case (D) (as analyzed in Section 3.1, Section 3.2, Section 3.3 and Section 3.4) is compared to a non-deforming case (R). To achieve the R case motion, the skeletal joints for the wing model (see Section 2.1) are altered such that the local joint rotations in the direction of the span and chord are 0, which removes the twisting and bending of the wing. We preserve the rigid pitching and rolling motion of the wing due to the root joint rotations. The difference in the kinematics is qualitatively and quantitatively compared in Figure 12.

In Figure 12a, wing chord lines are shown in dotted lines for the deformed wing (D) and solid lines for the rigid wings (R), which helps to identify discrepancies in twisting of the wings. Twisting is seen for the D case but not for R. Figure 12b compares the time series history of the wing *θ_twist_* at 0.75 R of the IW and OW for the R and D model. This quantitatively shows that where twist deformation for the D model is quite high, the R model has essentially zero deformation. In addition, plots of the average wing kinematics are compared for the cases to demonstrate the effect that the rigid condition has on the stroke, deviation, and angle of incidence. Some differences in kinematics are expected; after all, the tip trajectory is impacted by the lack of bending and twisting (as seen in Figure 12a). Figure 12c compares the wing kinematics for the R and D model, with the solid lines representing the whole stroke mean for the six flapping motions, and the shaded regions representing ± 1 s.d. from the mean. The stroke and deviation remain relatively similar, with the largest stroke difference occurring at the stroke reversal. Force generation at the stroke reversal is low, as observed in Figure 6, so we do not anticipate this leading to an extreme impact on the lift or drag forces. The AOI is quite different, as expected since the twisting angle of the wing directly impacts the computation of the LSRP on which the AOI calculation relies.

**Figure 13:**
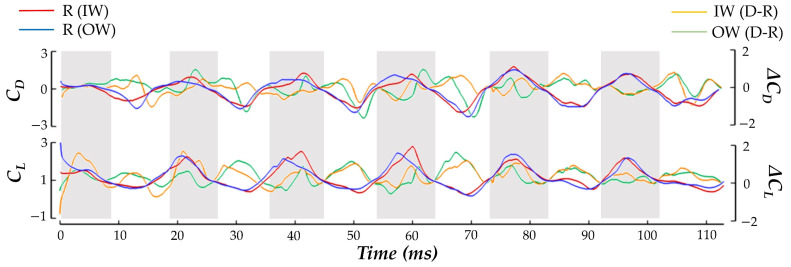
Drag and lift coefficient production comparison for the R model, with red indicating IW and blue indicating OW. The left-hand side y-axis represents the R model force coefficients, while the right-hand side y-axis represents the difference between the D and R model.

The R model force production is shown in Figure 13. Major differences between R and the D model are clear. Referring to the lift, an extended period of lower lift during the upstroke for both wings is produced by the IW and OW for the R model, a trend that is not observed for the D model. The ∆*C_L_*, shown in orange and green, show this difference as the D-R lift difference is shown to be quite large in the US (with the positive sign indicting D model lift is greater). This indicates that the wing deformations lead to more suitable generation of upwards lift throughout the turning motion to maintain a steady vertical elevation as observed in Section 3.1. Additionally, the drag force produced by the IW and OW is more symmetric for throughout the six strokes in the maneuver. This contrasts the highly asymmetrical drag generation for phase I and II that is exhibited by the D model. Particularly during the US periods we observe that the D-R drag difference for the OW is quite large, with the negative sign indicating that the R model drag is greater. Where the OW drag generation is less on the D model, permitting the drag asymmetry-induced torque, the R model OW generates nearly the same amount of force as the IW. This reveals that flexibility helps the hummingbird to achieve the drag force asymmetry necessary to complete the desired free yaw turn.

**Figure 14:**
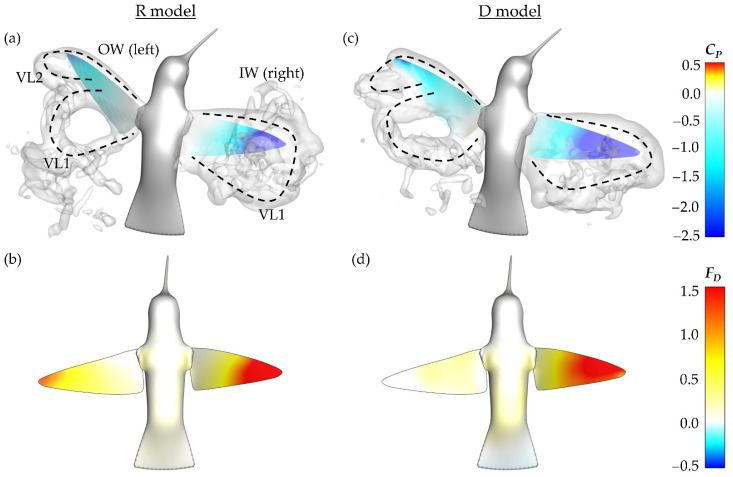
R model (**a**) surface pressure contours with pressure iso-surfaces at stroke 3 t/T* = 0.32 with corresponding (**b**) drag force production. (**c**,**d**) represent the same information for the F model.

Examining the surface pressure contour and pressure field at the time of maximum DS force production in stroke 3, along with the instantaneous drag force contours, some insight into the cause of the force symmetry is gained. The pressure field is visualized by iso-surfaces that indicate the non-dimensional pressure coefficient of −1.0. Figure 14a,b depict this information for the R model while Figure 14c,d are for the D model.

Firstly, it should be noted that the vortex formation, namely to dual-loop structures, is similar between both the rigid and flexible wing models. This is reflected by the generally close geometric organization of the pressure iso-surfaces for the R model in Figure 14a and D model in Figure 14c. As with Figure 8 and Figure 11, the VLs are labeled. Due to the lack of twist deformation, the IW for the R model exhibits a smaller region of low surface pressure in comparison to the D model. From the R and D model IW drag production in Figure 14b,d, it is observed that the R model generates correspondingly lower drag.

The OW for both models demonstrate very similar surfaces pressure contours. Despite this, the R model drag production on the OW is considerably larger than that of the F model. Comparing the drag production contours for the OW in Figure 14b,d, the R model has a concentration of drag production near the tip and relatively consistent, but lower, drag production across the rest of the span. The D model does not demonstrate this same trend; rather, the OW generates nearly no drag force at this particular time. The combination of weakened IW and increased OW drag production explains the general trend of the R model having more symmetric drag forces than the D model. Vortex formation between the rigid and twisting models is quite similar; however, wing flexibility helps to enhance the drag force asymmetry necessary to complete the maneuver in just six wingbeats (as with the D case) while also producing sufficient lift.

## 4. Conclusions

In this study, high-fidelity surface reconstruction techniques were combined with CFD simulations to study the pure yaw maneuver flight of a hummingbird. During the turn initiation phase, large differences in stroke, pitch, deviation angles, and twisting deformations are observed. Subsequently, during the turning phase, the hummingbird maintained a larger pitch and stroke amplitude for the IW along with larger twisting angles. The IW’s stroke, pitch, and twist angles were found to be about 13%, 16%, and ≤47% greater than those of the OW, respectively. Compared to coaxed-turn kinematics, the free maneuvering hummingbird in the current study turns at a greater speed, with suppressed US stroke angle and expanded DS deviation angle. To produce the pure yaw maneuver at such high turn rates, as in the studied free maneuvering hummingbird, MAV designs may take inspiration from these previously unknown free maneuvering wing kinematic characteristics.

These documented wing motions induce the force asymmetry and wake topology established by the simulation results. The drag force magnitude for the IW is 16% greater than the OW, with the US contributing about 63% more drag force than the DS. Meanwhile, lift generation between the two wings was similar, with the DS contributing about 65% of the total lift force. The drag force asymmetries can be attributed to the dual-vortex loop formation that occurs for the OW during the DS and IW/OW during the US. The dual-vortex loops form due to primary LEV instability, with separation from the leading-edge propagating in the wing tip to root direction. This phenomenon occurs early in the DS for the OW and US for the IW, leading to large VL2 formations in addition to the initially generated VL1. During the formation of the vortex loops, LEV strength discrepancies between the IW and OW (up to 59% greater circulation at peak DS force) are reflected in the relative size of low-pressure regions generated by the wings). Further, comparison of the low-pressure regions helps to explain the tendency for the force asymmetry to encourage the yaw turn in the US and damp the yaw turn in the DS. Understanding of this alternating torque generation role may be useful in MAV design. The ability to start, perform, and stop a turn within a few wingbeats, as demonstrated in the current study, is highly desirable and takes advantage of such counter-torque generation.

Finally, by analyzing a hummingbird model with rigid wings, the importance of flexibility in designing a MAV capable of performing a hummingbird-like yaw turn is clear. The lack of flexibility leads to more symmetric drag force production, which would reduce the yaw performance. Additionally, upwards lift was not produced at all stages of the flapping cycles suggesting that level flight may be more difficult or not achievable. This insight into flexibility, paired with the wing kinematics, force, and wake topology findings, represent a new understanding on biological and bio-inspired maneuvering. The kinematic and aerodynamic characteristics uncovered in the present work have the potential to inspire new generations of highly maneuverable hummingbird-like MAVs that mimic the stunning acrobatic motions of their biological counterparts.

## Figures and Tables

**Table 1:** Morphological data for calliope hummingbirds (*Stellula calliope*; *n* = 4).

Variable	Value
Body mass (g)	2.6 ± 0.2
Single wing length (mm)	40 ± 3
Wingspan, R (mm)	98 ± 1
Average wing chord, c (mm)	12 ± 1
Aspect ratio	3.3 ± 0.24
Single wing area (mm^2^)	505 ± 63
Disc loading (N m^−2^)	5.3 ± 1.1
Values are mean ± s.d. (*n* = 4)	

**Table 2:** Comparison of angular wing kinematics amplitude.

		Phase I	Phase II	Phase III
Stroke *Ψ_w_*	IW	96°	113 ± 5.3°	105°
	OW	114°	100 ± 6.9°	101°
Deviation *a_ww_*	IW	40°	40 ± 8.0°	33°
	OW	57°	48 ± 1.5°	35°
Pitch *Φ_w_*	IW	85°	97 ± 7.4°	120°
	OW	72°	85 ± 13.6°	110°

## Data Availability

Not applicable.
